# An Ecological Basis for Dual Genetic Code Expansion in Marine Deltaproteobacteria

**DOI:** 10.3389/fmicb.2021.680620

**Published:** 2021-07-14

**Authors:** Veronika Kivenson, Blair G. Paul, David L. Valentine

**Affiliations:** ^1^Interdepartmental Graduate Program in Marine Science, University of California, Santa Barbara, Santa Barbara, CA, United States; ^2^Department of Earth Science and Marine Science Institute, University of California, Santa Barbara, Santa Barbara, CA, United States

**Keywords:** microbiome, pyrrolysine, metabolism, selenocysteine, trimethylamine, methylotrophic

## Abstract

Marine benthic environments may be shaped by anthropogenic and other localized events, leading to changes in microbial community composition evident decades after a disturbance. Marine sediments in particular harbor exceptional taxonomic diversity and can shed light on distinctive evolutionary strategies. Genetic code expansion is a strategy that increases the structural and functional diversity of proteins in cells, by repurposing stop codons to encode non-canonical amino acids: pyrrolysine (Pyl) and selenocysteine (Sec). Here, we report both a study of the microbiome at a deep sea industrial waste dumpsite and an unanticipated discovery of codon reassignment in its most abundant member, with potential ramifications for interpreting microbial interactions with ocean-dumped wastes. The genomes of abundant Deltaproteobacteria from the sediments of a deep-ocean chemical waste dump site have undergone genetic code expansion. Pyl and Sec in these organisms appear to augment trimethylamine (TMA) and one-carbon metabolism, representing an increased metabolic versatility. The inferred metabolism of these sulfate-reducing bacteria places them in competition with methylotrophic methanogens for TMA, a contention further supported by earlier isotope tracer studies and reanalysis of metatranscriptomic studies. A survey of genomic data further reveals a broad geographic distribution of a niche group of similarly specialized Deltaproteobacteria, including at sulfidic sites in the Atlantic Ocean, Gulf of Mexico, Guaymas Basin, and North Sea, as well as in terrestrial and estuarine environments. These findings reveal an important biogeochemical role for specialized Deltaproteobacteria at the interface of the carbon, nitrogen, selenium, and sulfur cycles, with their niche adaptation and ecological success potentially augmented by genetic code expansion.

## Introduction

Marine sediments harbor a diverse and dense microbiome (Sogin et al., 2006; Orcutt et al., 2011) that has evolved over billions of years to perform a complex network of biogeochemical processes key to the carbon, nitrogen, selenium, and sulfur cycles. These environments now also serve as a repository for industrial, military, and municipal waste (Council on Environmental Quality, 1970; Duedall et al., 1983), which are expected to impart localized ecological and evolutionary pressures. One form of adaptation relevant to marine microorganisms is genetic code expansion (GCE), a type of code flexibility in which protein synthesis occurs with more than the twenty canonical amino acids. Known variants code for the 21st and 22nd amino acids, selenocysteine (Sec) and pyrrolysine (Pyl), respectively, through the repurposing of stop codons.

In bacteria, Sec is inserted in polypeptides at an in-frame UGA (opal/stop) codon, via recognition of a downstream ∼50 nt hairpin structure known as a Sec insertion sequence (Zinoni et al., 1990; Liu et al., 1998). Proteins containing Sec occur in a subset of organisms in each of the three domains of life, including many bacteria such as members of Proteobacteria, Chloroflexi, and Firmicutes (Böck et al., 1991; Low and Berry, 1996; Böck, 2000). Sec is part of the active site of a subset of redox enzymes involved in energy metabolism, conferring a higher catalytic efficiency (Cone et al., 1976; Zinoni et al., 1987; Mukai et al., 2016); for example, when Sec is replaced by its counterpart, cysteine, in formate dehydrogenase, the rate of oxidation is slowed by approximately three orders of magnitude (Axley et al., 1991). These proteins have critical roles in marine biogeochemical cycles (Copeland, 2005; Zhang and Gladyshev, 2008) and microbial Sec- utilization may be facilitated in aquatic/marine environments because of a sufficient supply of the required element, Selenium, in the ocean (Peng et al., 2016).

Pyrrolysine is encoded in place of an in-frame UAG (amber/stop) codon and occurs in <1% of sequenced organisms- primarily methanogenic archaea (Atkins and Gesteland, 2002; Srinivasan et al., 2002; Gaston et al., 2011a). The Pyl residue is crucial as part of the active site of mono-, di-, and tri- methylamine methyltransferases (Paul et al., 2000; Atkins and Gesteland, 2002; Gaston et al., 2011b). Methylated amines are ubiquitous in marine sediment, the water column, and marine aerosols, and play a significant role in ocean carbon and nitrogen cycles (Sun et al., 2019). Trimethylamine (TMA) is an important compound in this family, with concentrations ranging from a few nM in the water column, to low μM in sediment pore water, representing a significant pool of dissolved organic nitrogen (Chen, 2012; Sun et al., 2019). Along with their precursor quaternary amines, methyl amines contribute significantly to the production of methane (Borges et al., 2016).

Although anaerobic metabolism of methylated amines is commonly attributed exclusively to methanogenic archaea, genetic code expansion with Pyl was identified in TMA-utilizing bacteria, including *Acetohalobium arabaticum* (Prat et al., 2012), human gut bacteria (Kivenson and Giovannoni, 2020), and uncultivated marine worm symbionts that also encode Sec (Zhang and Gladyshev, 2007). However, the occurrence, distribution, and metabolic significance of genetic code expansion in bacteria from marine environments remains unknown.

In this study, we capitalize on our recent discovery of a deep ocean industrial waste dump (Kivenson et al., 2019) to investigate the perturbed sediment microbiome and examine genetic code expansion which may have significance in marine sediments at this site and in other benthic environments. In the dominant Deltaproteobacteria, we find extensive evidence of a largely unexplored mechanism for TMA and one-carbon metabolism augmented by dual genetic code expansion. We characterize these Sec- and Pyl- dependent metabolic pathways and identify additional occurrences of these adaptations in widely distributed Deltaproteobacteria from genomic, metagenomic, and transcriptomic data, uncovering an important role for these organisms in coupling the carbon, nitrogen, selenium, and sulfur cycles in oxygen-limited marine environments.

## Results and Discussion

### An Industrial Waste Microbiome

**FIGURE 1:**
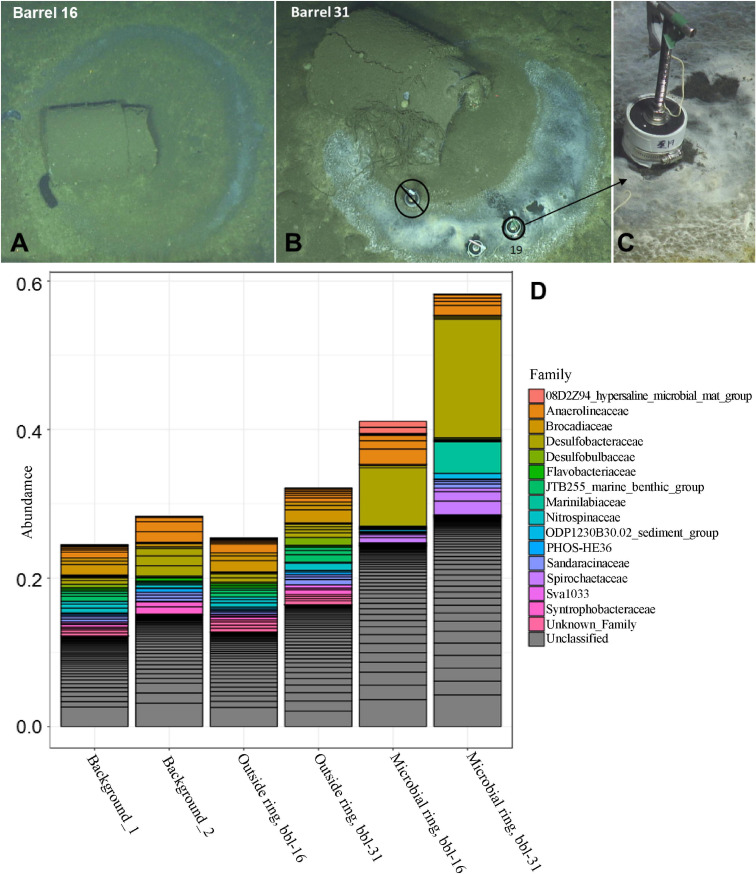
**(A)** Barrel 16 encircled by a microbial mat ring **(B)** Barrel 31 with microbial mat ring; sediment collection site of Core 19. **(C)** Close-up of Core 19 at barrel 31 microbial mat **(D)** Abundance of top taxa by sample for background, outside ring, and microbial ring samples.

Photo-surveys using autonomous and remote underwater vehicles revealed approximately sixty of an estimated half a million barrels dumped in the San Pedro Basin, off the coast of California (Supplementary Figure 1), as described in our previous study (Kivenson et al., 2019). A subset of the barrels exhibited a ring-form microbial mat that commonly, but not exclusively, encircled the barrels. Burrows were present throughout the seafloor and notably absent between each barrel and its microbial mat ring (Supplementary Figure 2). A survey of 16S rRNA genes was conducted for the upper 2 cm of sediment at the microbial mats, just outside of these mats, and ≥60 m from any visible barrels, to determine the identity and relative abundances of microbial taxa associated with these habitats. The circular mat rings were sampled at barrel 16 (bbl-16) (Supplementary Figure 3 and Figure 1A), and barrel 31 (bbl-31) (Figures 1B,C). These features were less diverse than surrounding sediments (Figure 1D and Supplementary Figure 4), consistent with a response to a major environmental disturbance.

The most abundant taxa for both of the microbial mat samples (ASV-1) is a member of the *Desulfobacteraceae* family, most closely affiliated with the *Desulfobacula* genus, and comprises 16% and 8% of the total bacterial community for bbl-31 and bbl-16, respectively (Figure 1D and Supplementary Table 1). These abundances are substantially greater than that of the most abundant organism, a member of the SEEP-SRB1 clade of *Desulfobacteraceae*, comprising ∼3% of the microbial community in surface sediment at the nearby San Pedro Ocean Times Series (SPOTS) station (Monteverde et al., 2018). As observed in the SPOTS sediment dataset, sediments (non-ring samples) did not have a single taxa exceeding 4% of the total community (Supplementary Table 1).

### Genome Reconstruction of Enriched Taxa

Metagenomic sequencing was performed for the six sediment samples, with between 9 and 22 GB of raw sequencing reads generated per sample (Supplementary Table 2). In total, eleven genomes were reconstructed from the two microbial mat samples, of which six are estimated to be >90% complete (Table 1 and Supplementary Tables 3, 4). The reconstructed genomes include members of the *Desulfobacterales, Bacteroidales, Gemmatimonadetes, Victivallales*, and *Spirochaetales* orders, as well as several candidate phyla: *Zixibacteria, Woesearchaeota, Marinimicrobia*, and *Latescibacteria*. Many of these taxa are commonly detected in oxygen-limited marine sediment, including the SPOTS data set (Monteverde et al., 2018).

**TABLE 1:** Reconstructed genome statistics including genome size, mean coverage, GC content, estimated percent completion and redundancy, and taxonomic classification.

Reconstructed genomes	Size (Mb)	Mean Coverage	GC%	Completion	Redundancy	Taxonomy (Order unless otherwise specified)
CORE_8_RING_Bin_00001 **(Deltaproteobacteria-bbl-16)**	2.55	83	41	97%	1%	Desulfobacterales
CORE_8_RING_Bin_00002	3.28	23	50	94%	0%	Candidate division Zixibacteria (Phylum)
CORE_8_RING_Bin_00003	2.27	13	39	86%	0%	Bacteroidales
CORE_8_RING_Bin_00004	0.61	21	28	50%	0%	Candidatus Woesearchaeota (Phylum)
CORE_8_RING_Bin_00005	2.50	12	44	42%	4%	Candidatus Marinimicrobia (Phylum)
CORE_19_RING_Bin_00001 **(Deltaproteobacteria-bbl-31)**	2.82	123	41	99%	1%	Desulfobacterales
CORE_19_RING_Bin_00002	3.39	45	32	97%	4%	Bacteroidales
CORE_19_RING_Bin_00003	3.78	15	51	93%	0%	Candidatus Latescibacteria (Phylum)
CORE_19_RING_Bin_00004	3.58	36	56	93%	3%	Gemmatimonadetes
CORE_19_RING_Bin_00005	1.74	10	40	75%	0%	Victivallales
CORE_19_RING_Bin_00006	2.13	13	58	66%	1%	Spirochaetales

Consistent with the analysis of diversity, the four sediment samples not associated with a microbial mat resulted in limited assembly as evidenced by metrics such a low N50, low mean contig length, and low total assembly length (Supplementary Table 2), and genome reconstruction was not viable for these samples.

### Deltaproteobacteria Dominate Sediments Underlying Microbial Mats

A near-complete *Deltaproteobacteria* genome most closely related to members of the *Desulfobacula* genus was recovered from the sediments underlying the microbial mat at bbl-31. The mean coverage of this genome is 123x—the highest observed from the sample – indicating that this is the most abundant mat organism (Supplementary Table 3). An 1109 bp fragment of the 16S rRNA gene recovered from the reconstructed genome aligns with the most abundant 16S rRNA ASV (ASV-1), indicating a consensus between the 16S rRNA and metagenomic data sets, and confirming that the genome of the most abundant organism at the mat was identified and reconstructed. The 16S rRNA gene sequence has a percent identity of 94.4% with the nearest (*Desulfobacula*) match, indicating that this organism has a marginal affiliation with this genus (a cut-off of ≥94.5% across a full-length sequence would indicate genus-level membership).

The most abundant genome recovered from sediment at the bbl-16 microbial mat (83x mean coverage) is also a member of *Deltaproteobacteria*, and the average nucleotide identity (ANI) of these two Deltaproteobacteria bbl genomes is 99%. The genomes described here will be referred to as Deltaproteobacteria-bbl-31 and Deltaproteobacteria-bbl-16 based on the identity of the proximal waste barrel. In addition to the ANI, the predicted genome size, GC content, and multiple other features of these two genomes are highly similar (Supplementary Tables 3, 5). These findings indicate reproducibility in the microbial response to waste dumping, at two different locations.

### Properties of Dominant Deltaproteobacteria

The near-complete *Deltaproteobacteria-bbl-31* genome size is ∼2.6 Mbps, about half the size of cultivated members of the related *Desulfobacula* genus, which includes several described members: *Desulfobacula toluolica* (Wöhlbrand et al., 2013), *Desulfobacula phenolica* (Kuever et al., 2001), and *Desulfobacula sp. TS* (Kim et al., 2014). Additional related Deltaproteobacterial genomes from public databases were identified based on a high percent identity of ribosomal proteins, and are from hydrothermal vent sediments (Dombrowski et al., 2017, 2018) and aquifer groundwater (Castelle et al., 2013). These environmental genomes are similar in size to those from this study and share many of the metabolic traits, discussed in detail in the next section. The genome that has the highest percent identity for the ribosomal proteins is Desulfobacteraceae bacterium 4572_123 from the Guaymas Basin, and the proteins from this genome and those of Deltaproteobacteria-bbl genomes have a two-way average amino acid identity (AAI) of 52.16% (the AAI genus threshold is 55–60%) (Rodriguez-R and Konstantinidis, 2014) suggesting that these two organisms are related, and both belong to the *Desulfobacteraceae* family.

**FIGURE 2:**
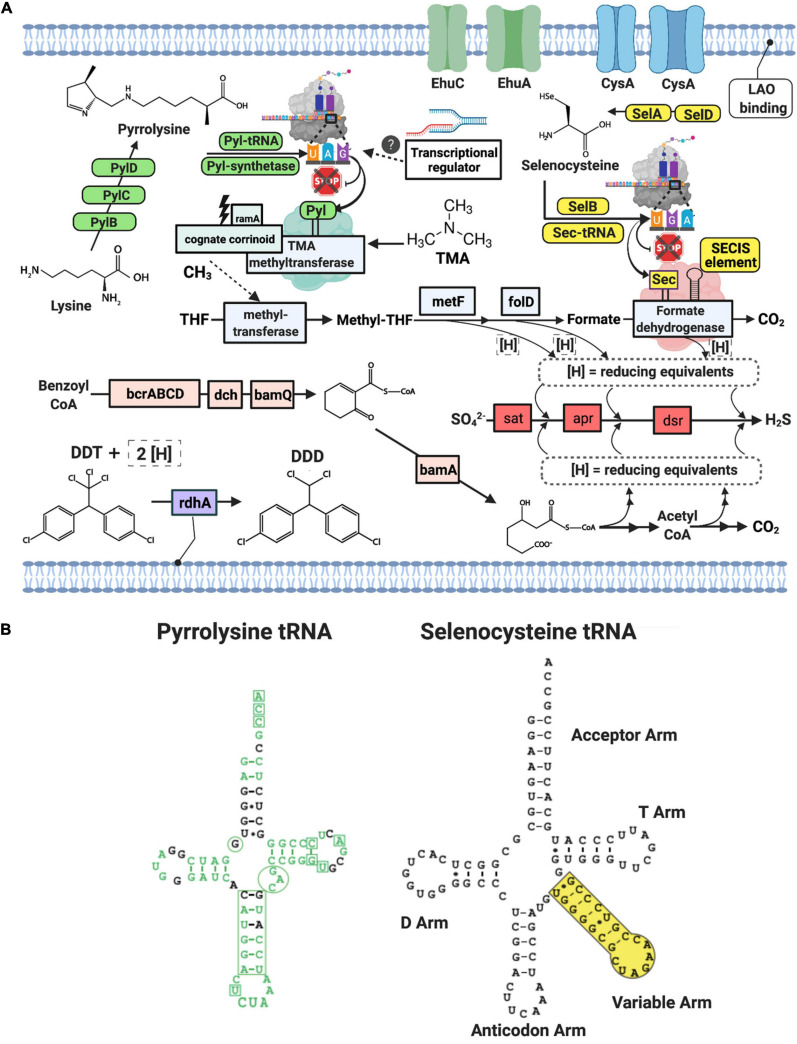
**(A)** Schematic of Deltaproteobacterial-bbl cell and metabolic potential with green and yellow icons indicating machinery necessary for genetic code expansion for Pyl and Sec, respectively. Blue icons indicate proteins in the Wood-Ljungdahl pathway, pink icons indicate proteins in the benzoyl CoA pathway, and light purple is involved in dehalogenation. Gene and compound abbreviations: PylB, 3-methylornithine synthase; PylC, 3-methylornithine–L-lysine ligase; PylD, 3-methylornithyl-N6-L-lysine dehydrogenase; Pyl synthetase, pyrrolysyl-tRNA synthetase; Pyl tRNA, pyrrolysine transfer RNA; ramA, methylamine methyltransferase corrinoid protein reductive activase; cognate corrinoid, methyltransferase cognate corrinoid protein; SelD, selenide, water dikinase; SelA, L-seryl-tRNA(Sec) selenium transferase; SelB, selenocysteine-specific translation elongation factor, Sec-tRNA, selenocysteine transfer RNA; SECIS Element, selenocysteine insertion sequence element; EhuC, ectoine hydroxyectoine ABC transporter C; EhuA, ectoine hydroxyectoine ABC transporter A; LAO binding, lysine-arginine-ornithine binding periplasmic protein, rdhA, reductive dehalogenase; DDT, Dichlorodiphenyltrichloroethane; DDD, dichlorodiphenyldichloroethane; THF, tetrahydrofolate; metF, methylenetetrahydrofolate reductase; folD, methylene-tetrahydrofolate (CH2-THF) dehydrogenase/cyclohydrolase; bcrABCD, benzoyl CoA reductase subunits A,B,C,D; dch, dienoyl-CoA hydratase; bamQ, 6-hydroxycyclohex-1-ene-1-carbonyl-CoA dehydrogenase; bamA, 6-oxocylcohex-1-ene-1-carbonyl-CoA hydrolase; sat, sulfate adenylyltransferase; apr, adenylylsulfate reductase, dsr, dissimilatory sulfite reductase subunit alpha and beta. **(B)** Secondary structures of the pyrrolysine tRNA and selenocysteine tRNA from Deltaproteobacteria-bbl genomes. Green text indicates match with Methanosarcina tRNA sequence, including the corresponding CUA anticodon. Additionally, the boxes and circles show locations of highly conserved regions of the Methanosarcina pyrrolysine tRNA. Small black dots in stems indicate wobble base pairs.

The metabolic potential for the Deltaproteobacteria-bbl genomes from this study largely overlaps with that of the *Desulfobacula*, and these genomes encode the full pathway for dissimilatory sulfate reduction, including the sulfate adenylyltansferase, adenylylsulfate reductase, and dissimilatory sulfite reductase proteins (Figure 2A). Similar to members of the *Desulfobacula* genus, these organisms also harbor the capacity for xenobiotic compound degradation, and have the marker gene for anaerobic aromatic degradation, bamA (6-oxocyclohex-1-ene carbonyl CoA hydrolase) (Porter and Young, 2013), and the marker gene for organohalide respiration, rdhA (reductive dehalogenase) (Holliger et al., 1998; Hug et al., 2013) (Figure 2A). Additionally, the genes for the Benzoyl-CoA to Acetyl-CoA pathway are also encoded, although the metabolic precursor to Benzoyl-CoA remains undetermined in this genome (Figure 2A). These anaerobic aromatic degradation and dehalogenation abilities may be beneficial for this organism in the environment, given the nature of anthropogenic waste (e.g., chlorinated aromatic hydrocarbon compounds) present at this study site (Kivenson et al., 2019). While the persistence by members of the genus at high abundance could suggest they can at least tolerate these toxins, further experiments would be required to address specific genetic or metabolic mechanisms by which tolerance/detoxification may occur.

### Metabolic Augmentation by Genetic Code Expansion

Natural genetic code expansion is defined as the ability to encode more than twenty canonical amino acids. The Deltaproteobacteria bbl-31 genome has the full set of cellular machinery required for both naturally occurring non-canonical amino acids. For Sec utilization in bacteria, a Sec- specific tRNA (selC) is required, as well as several proteins: Sec- synthase [alternatively termed L-seryl-tRNA (Sec) selenium transferase; selA], a Sec-specific translation elongation factor (selB), and selenophosphate synthetase (alternatively termed selenide water dikinase; selD, which itself contains a Sec residue) (Stadtman, 1996; Figure 2A). Along with the Sec- specific machinery proteins, the Sec- tRNA allows the cell to co-translationally insert Sec by repurposing the UGA, or opal codon (which would otherwise signal a stop), in instances when an adjacent, downstream SECIS element (a ∼50 nt hairpin structure) is present. In the Deltaproteobacteria-bbl genomes (and other Sec-utilizing organisms), the Sec- tRNA differs from most other tRNA: instead of a four-nucleotide variable loop, this tRNA has a variable arm between the anticodon arm and the T arm (Figure 2B).

Selenium (Se) is transported into cells in the form of the oxyanions, selenate (SeO_4_^2–^) and selenite (SeO_3_^2–^). Because sulfur and Se are analogs, the transport of Se oxyanions occurs via sulfate transporters (Lindblow-Kull et al., 1985; Turner et al., 1998). In each of the Deltaproteobacteria-bbl genomes, there are two copies of the sulfate/thiosulfate import ATP-binding protein, CysA (TIGR00968). These are adjacent to other transport-related proteins which may also be involved in S or Se transport, although a single transporter is sufficient for Se transport in *Escherichia coli* (Lindblow-Kull et al., 1985).

In addition to the specialized Sec machinery, the incorporation of Sec in the cell is further evidenced by the presence of an in-frame UGA codon followed by the SECIS element in a subset of its genes. In Deltaproteobacteria-bbl-31, these features occur in a total of 34 putative selenoproteins, belonging to two groups (Supplementary Tables 6, 7). The first group encodes Sec in a conserved position of previously characterized selenoproteins, including those described from marine environments (Zhang et al., 2005; Zhang and Gladyshev, 2007). This group is comprised largely of redox related proteins, and in the Deltaproteobacteria-bbl genome, it includes formate dehydrogenase (Figure 2A), coenzyme F_420_-reducing hydrogenase, methyl-viologen-reducing hydrogenase, sulfurtransferase, a domain of unknown function (DUF 166), Fe-S oxidoreductase, heterodisulfide reductase, a rhodanese-like domain, and the selenophosphate synthetase (Supplementary Table 6). There are multiple copies of genes encoding a subset of these Sec- containing proteins. The second group is comprised of novel, potential selenoproteins, identified using a published *in silico* recognition algorithm (Zhang and Gladyshev, 2005) for locating in-frame UGA residues followed by the SECIS element. These putative new selenoproteins include endonuclease III, cobinamide phosphate guanylyltransferase, a histidine kinase, and others (Supplementary Table 6). Although further verification (i.e., confirmation of expression) is needed to confirm the incorporation of Sec and subsequent activity of these proteins, these observations suggest an increased range of proteins in which Sec may occur. Furthermore, while Sec is known to increase the catalytic efficiency of proteins, its presence in multiple non-redox proteins suggests that it has additional, as yet unknown role(s).

**FIGURE 3:**
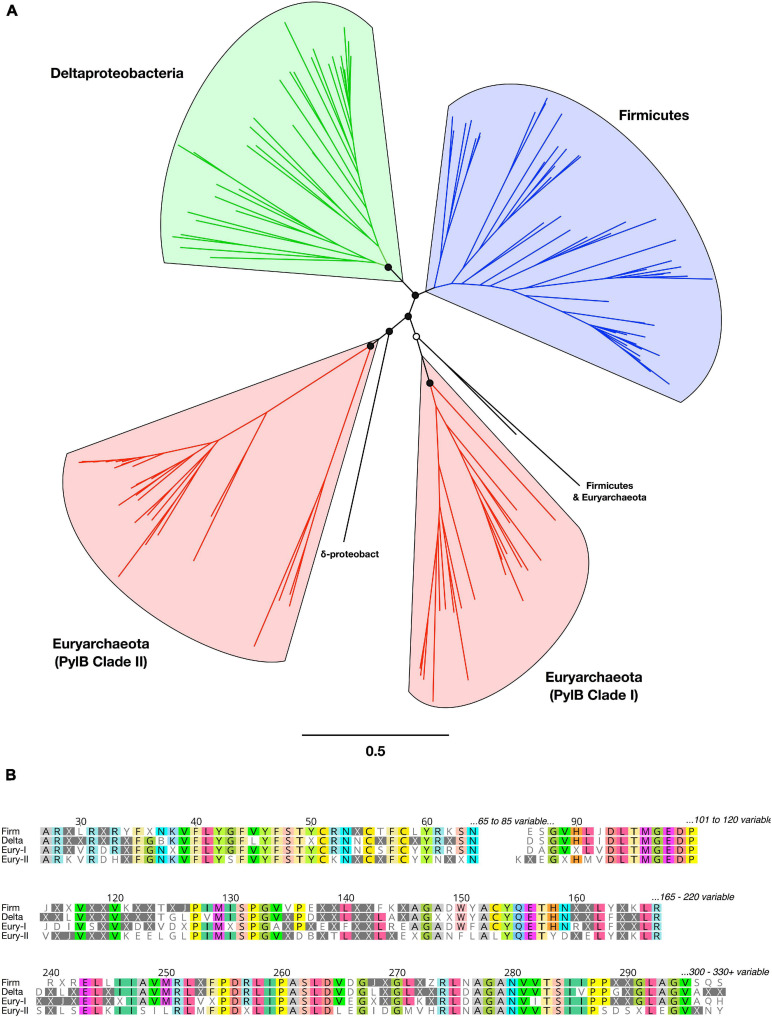
Phylogeny, diversity, and conserved regions of PylB proteins in Bacteria and Archaea. **(A)** Unrooted phylogenetic tree from PylB sequence alignment. Major clades are highlighted in color by taxonomic classification. Branch support is shown for the basal clades only (open circles >50%; filled circles >75%). All tip labels and branch support values are provided in Supplementary Figure 5. **(B)** Conserved regions from an alignment of PylB consensus amino acid sequences representing four major clades. A consensus sequence was generated for each of the four PylB clades with a threshold of >50% identical amino acids found in a clade; positions with <50% agreement are indicated with “X.” Conserved positions in the alignment are shown and amino acids are highlighted with a >75% identity threshold.

The rare amino acid, Pyl, is synthesized from two molecules of L-lysine via the action of three proteins: 3-methylornithine synthase (pylB), 3-methylornithine-L-lysine ligase (pylC), and 3-methylornithyl-N6-L-lysine dehydrogenase (pylD) (Gaston et al., 2011b). We analyzed phylogeny and taxonomic diversity for putative PylB protein sequences obtained here, in comparison with other homologs from bacterial and archaeal genomes (Figure 3 and Supplementary Figure 5). Four monophyletic clades were delineated by taxonomy, but several divergent sequences were separately identified from Firmicutes, Deltaproteobacteria, and Euryarchaeota genomes. Examination of the alignment of all PylB-like sequences revealed conserved regions shared across the four distinct clades (Figure 3B). A radical SAM domain (PF04055) was predicted in most sequences between residues ∼50 to ∼210. All sequences have homology to the pyrrolysine biosynthesis protein PylB family (IPR023891; also homologous to IPR034422 and IPR024021).

Co-translational insertion of Pyl into the growing polypeptide at an in-frame UAG (amber) codon requires Pyrrolysyl-tRNA synthetase, and a Pyl- specific tRNA (Srinivasan et al., 2002; Polycarpo et al., 2004; Gaston et al., 2011b). Along with the Pyl synthesis proteins and synthetase, the specialized amber suppressor tRNA is encoded in the Deltaproteobacteria-bbl genomes (Figure 2A). Originally identified in the *Methanosarcina* genus, this Pyl- tRNA has an atypical secondary structure, including a hallmark CUA anticodon, an anticodon stem with an extra nucleotide, and one less nucleotide between the acceptor and D arm in the secondary structure (Srinivasan et al., 2002). These unusual features are evident in the secondary structure of Pyl-tRNA in the Deltaproteobacteria-bbl genomes, which show a high degree of conservation with the Methanosarcina Pyl- tRNA (Figure 2B). Earlier studies indicate that incorporation of Pyl is required as part of the active site of mono-, di-, and tri- methylamine methyltransferases (Paul et al., 2000; Hao et al., 2002; Srinivasan et al., 2002). Both Deltaproteobacteria-bbl genomes have TMA methyltransferase with a conserved active site containing the Pyl residue encoded by an in-frame amber codon at position 327 of 485 (similar to that in archaeal methanogens, which have Pyl at position 334 of 483) (Paul et al., 2000).

In methanogens, the methyl from TMA is transferred by the TMA methyltransferase to a cognate corrinoid protein (Paul et al., 2000). Before accepting the methyl group, the corrinoid protein cobalt (II) active site is reduced to cobalt (I) by a ferredoxin-like protein with an Fe_4_S_4_ domain, known as a methylamine methyltransferase corrinoid protein reductive activase (ramA) (Ferguson et al., 2009). Next, the methyl group is transferred to methyl-coenzyme M (Methyl-CoM) via a second methyltransferase (coenzyme M methyltransferase, MtbA, which acts as the common CoM methylase for MMA, DMA, and TMA) and methanogenesis can proceed by the action of methyl coenzyme M reductase (mcrA) (Wolfe and McBride, 1971; Ferguson et al., 1996; Burke et al., 1998). With the exception of the MtbA and mcrA, the Deltaproteobacteria-bbl genomes encode the aforementioned genes for methyl utilization (Figure 2A).

In the Deltaproteobacteria, an alternate path may be utilized, wherein the methyl group is transferred to tetrahydrofolate (THF) and enters the Wood-Ljungdahl (WL) pathway. In bacterial systems with methyl transfer to THF, a complex composed of three proteins is utilized: a substrate specific methyltransferase, a corrinoid binding protein, and a second methyltransferase which methylates a cellular cofactor (Matthews, 2009; Picking et al., 2019). In the Deltaproteobacteria-bbl genomes, we have identified the Pyl-containing trimethylamine methyltransferase as the putative substrate specific methyltransferase (Supplementary Table 8). We further identified the cognate corrinoid protein (TIGR0237, pyl- corrinoid: methyltransferease cognate corrinoid protein, Methanosarcina family) encoded by a gene located nearby (<10 genes) (Supplementary Table 9). To date, the second methyltransferase of the bacterial TMA complex remains uncharacterized. The archaeal counterpart, MtbA, is homologous to methionine synthase (metH) (Paul and Krzycki, 1996), and is clustered with the TMA methyltransferase and cognate corrinoid (Burke et al., 1998). The Deltaproteobacteria-bbl-31 genome also encodes a metH family protein in this gene neighborhood (<10 genes) (Supplementary Table 9), and here we identify it as a potential second methyltransferase. The top template match for the predicted structure of this protein is the methyltransferase component involved in o-demethylation (100% confidence, 99% coverage, 39% id), wherein methyltetrahydrofolate is formed from aromatic methyl ethers (Sjuts et al., 2015). While the percent identity is low (39%), this result and the gene neighborhood is consistent with a methyltransferase role for the metH family protein. Additionally, some methyltransferase proteins are promiscuous in their methyl sources (Becher et al., 1992; Adam et al., 2018), so it is also possible that this protein functions as a component of (but is not specific to) the TMA system. By this proposed route, the cognate corrinoid would serve as the methyl acceptor from TMA and the methyl donor for THF, with trimethylamine methyltransferase and the metH family methyltransferase catalyzing the respective transfers.

The full set of WL pathway proteins is encoded in the Deltaproteobacteria-bbl genomes (Figure 2A). The WL pathway may operate in reverse (for carbon compound oxidation) in *Desulfobacula* and other sulfate reducing bacteria (Wöhlbrand et al., 2013), and be paired with sulfate reduction, and ultimately used for energy for the cell. Intriguingly, two of the key proteins in the proposed reverse WL pathway contain residues arising from independent instances of genetic code expansion: the Pyl-containing TMA methyltransferase and the Sec-containing formate dehydrogenase (Figure 2A). This dual genetic code expansion- augmented metabolism may be beneficial for the cell due to linking of advantageous traits: the utilization of a ubiquitously available compound (TMA) by the Pyl-containing methyltransferase, and the increased catalytic efficiency conferred by the Sec-containing formate dehydrogenase.

Additional evidence for the importance of genetic code expansion- augmented metabolism in the Deltaproteobacteria-bbl (and other Deltaproteobacterial) genomes is the presence of multiple genes relevant to Pyl synthesis and methyl amine metabolism, proximal to the TMA methyltransferase gene (Supplementary Table 9). This functional gene organization may have biological significance, as it is completely conserved within the Deltaproteobacteria-bbl genomes, and is highly similar in related genomes from other locations (discussed further in the following section on biogeographic distribution). In the *Deltaproteobacteria-bbl* and related genomes, the gene adjacent to TMA methyltransferase encodes a lysine-arginine-ornithine binding periplasmic protein, which may play a role in Pyl synthesis, as lysine is the precursor to pyrrolysine (Krzycki, 2013). Moreover, the addition of ornithine to growth media has been shown to increase the yield of full-length Pyl proteins (Namy et al., 2007). The following two genes encode ectoine/hydroxyectoine permeases/ABC transporters; ectoine and hydroxyectoine are compatible solutes and the aforementioned transporter class of proteins is involved in the transport of methylated amines into the cell (Burke et al., 1998). Also proximal to the methyltransferase gene is a transcriptional regulator, though a more specific functional role remains undetermined. In members of the *Firmicutes*, transcriptional feedback loops control the genetic code expansion and subsequent use of TMA via the TMA methyltransferase (Prat et al., 2012). A putative glycine betaine methyltransferase gene also occurs in this locus, which is commonly mis-annotated as a trimethylamine methyltransferase (Ticak et al., 2014), but does not encode pyrrolysine. In other organisms, the role of this protein is to convert glycine betaine to dimethylglycine and methylcobalamin (Ticak et al., 2014). Multiple copies of this non-pyrrolysine methyltransferase are present elsewhere in each of the *Deltaproteobacteria-bbl* genomes (Supplementary Table 10), suggestive of a broad capacity for methyl metabolism in these organisms.

### Deltaproteobacteria May Compete With Methanogens for TMA

**FIGURE 4:**
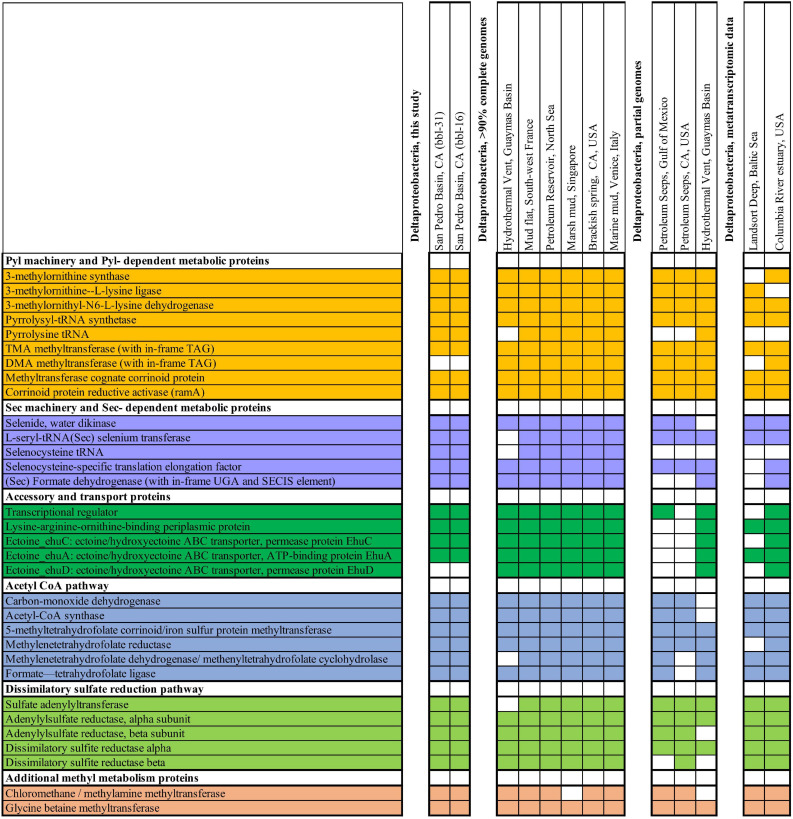
Genes from metabolic, biosynthetic, and other pathways related to genetic code expansion-enabled methyl utilization, shown from Deltaproteobacteria from this study along with other genomic and transcriptomic data sets. Colored boxes indicate that the gene is present in Deltaproteobacteria as either transcriptomic or genomic sequence.

**FIGURE 5:**
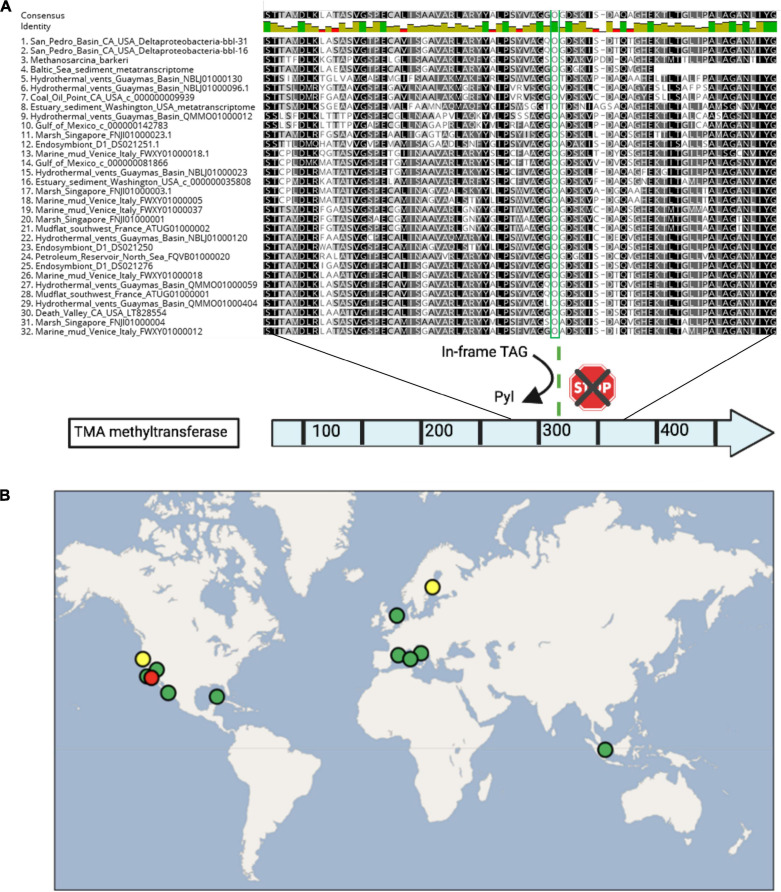
**(A)** Alignment of select residues of the trimethylamine methyltransferase protein, spanning the pyrrolysine residue (in green), as well as the conserved residues following the UAG codon. Proteins are shown from the Deltaproteobacteria-bbl genomes from this study, *Methanosarcina barkeri* for comparison, and Deltaproteobacteria from other studies, including two transcriptomics data sets. **(B)** The global distribution of dual genetic code expansion-enabled Deltaproteobacteria that have the trimethylamine methyltransferase and conserved Pyl residue, as well as Sec and Pyl machinery genes. Circle colors are as follows: red indicates this study, yellow indicates metatranscriptomic data sets, and green includes metagenomic/genomic data sets. The location source and type of each dataset is provided in Supplementary Table 11.

Based on the capacity for TMA methyl-group oxidation encoded by the Deltaproteobacteria-bbl-31 and Deltaprotebacteria-bbl-16, we mined published metagenomic and metatranscriptomic data from sulfidic sediments for indications of Pyl incorporation by *Deltaproteobacteria* (Supplementary Table 11). In reanalyzing a recent study of Baltic Sea sediment (Thureborn et al., 2016) we identified Deltaproteobacterial expression of the TMA methyltransferase, and of the Pyrrolysyl-tRNA synthetase, PylD and PylC proteins (Figure 4). Furthermore, the expressed TMA methyltransferase from the Baltic Sea transcriptomic dataset is nearly identical (96%) with that of the Deltaproteobacterial-bbl protein. Importantly, the position of the pyrrolysine is conserved, as well as the amino acid sequence following the UAG codon (Figure 5A).

These observations reveal that TMA metabolism enabled by genetic code expansion is actively utilized by Deltaproteobacteria in the environment, putting them in competition with methylotrophic methanogens. At a minimum, TMA use in sediment is not restricted to the archaea. While methanogens (primarily Methanosarcina) are active at the Baltic Sea site, there is no detectable expression of TMA (or DMA or MMA) –methyltransferase by any archaea. The primary expressed methanogenesis pathway is hydrogenotrophic (CO_2_ + H_2_), indicating that oxidation of TMA by Deltaproteobacteria may be an important environmental fate for this compound.

To further investigate the occurrence of TMA-utilizing Deltaproteobacteria, we analyzed an additional sediment metatranscriptome from the intertidal zone of the Columbia River estuary, Washington, United States where archaeal methylotrophs have been reported (Smith et al., 2015, 2019). Similarly to the Baltic Sea sediment, this environment hosts Deltaproteobacteria which express the pyrrolysyl-tRNA synthetase, PylB, PylC, and TMA methyltransferase spanning the Pyl-containing active site. In addition to expression of the Pyl- dependent TMA methyltransferase, the Deltaproteobacteria at this site express Sec machinery proteins, Sec-containing formate dehydrogenase and other one-carbon metabolic pathway proteins (Figure 4).

The co-occurrence of active TMA-utilizing Deltaproteobacteria and methylotrophic archaea in the Baltic Sea and Columbia River intertidal estuary samples may be due to the metabolic versatility of these organisms, the availability of sufficient quantities of TMA to support both groups, or niche partitioning, with the archaea potentially utilizing compounds produced by the Deltaproteobacteria.

While TMA in oxygen- limited environments has been referred to as a non-competitive substrate that is used exclusively by methanogenic archaea, for example (Reeburgh, 2007; Joye et al., 2009; Orsi et al., 2013; Kelley et al., 2014; Zhuang et al., 2016), the pioneering studies that first examined methylated amine utilization in anoxic sediments demonstrated that TMA consumption rates exceed their methanogenic potential and that bacteria also utilize methyl amines (King, 1984a,b). Moreover, addition of sulfate reduces the fraction of substrate metabolized to methane from trimethylamine in incubation experiments (Lovley and Klug, 1983). Similarly, a more recent study of TMA utilization in marine sediments demonstrated that inhibition of sulfate reducing bacteria increased the rate of TMA-methanogenesis (Xiao et al., 2018), and another study describes that TMA-amended incubations resulted in H_2_S production (Cadena et al., 2018), again pointing to competitive TMA utilization by anaerobic bacteria. Collectively, available genomic, transcriptomic and environmental data indicate that Deltaproteobacteria consume TMA using metabolism dependent on genetic code expansion, and may compete with archaeal methylotrophs in the environment.

### A Cosmopolitan Distribution for Deltaproteobacteria With Genetic Code Expansion- Augmented Metabolism

To assess the geographic distribution of *Deltaproteobacteria* with metabolism augmented by genetic code expansion, we surveyed publicly available Deltaproteobacteria genomes from marine and terrestrial environments for GCE genes (Supplementary Tables 11, 12). In addition to the genomes from this study, nine other *Deltaproteobacterial* genomes (six of which are ≥90% complete) were identified with the Pyl and Sec biosynthesis proteins (Figure 4). The Deltaproteobacteria from these studies also encode a TMA methyltransferase protein with a conserved Pyl residue (Figure 5A). These bacteria originate from diverse settings; in addition to the San Pedro Basin (this study), they include hydrothermal vents in the Guaymas Basin, marine mud in Italy, petroleum seeps in the North Sea and California, as well as terrestrial environments, including a marsh in Singapore and brackish spring in California (Figure 5B). Most of these Deltaproteobacteria have multiple copies of TMA methyltransferase (with up to five copies in a single genome) and the dimethylamine methyltransferase with an in-frame Pyl. Additionally, most of the genomes have all of the proteins necessary for the reverse WL pathway, including a Sec-containing formate dehydrogenase (Figure 4). These specialized bacteria inhabit many settings that are oxygen-limited and/or hydrocarbon-rich, much like the related *Desulfobacula* genus. Taken together, the distribution of these Deltaproteobacteria and their genomic repertoire indicates an important geochemical role governing the fate for TMA in the environment.

## Materials and Methods

### Site Description and Sampling

The study site is located at ∼894 m water depth in the oxygen-limited San Pedro Basin, off the coast of California, at 33.566N, 118.426W. It was accessed in October 2013 using the autonomous underwater vehicle (AUV) Sentry (dives S-202 and S-208) and the remotely operated vehicle (ROV) Jason (dive J2-746), during RV Atlantis Leg 26-06, as previously described (Valentine et al., 2016; Kivenson et al., 2019). Briefly, the AUV Sentry was deployed to locate the industrial waste dumpsite, and high-resolution multibeam echosounder bathymetry at 60 m above the seafloor indicated the presence of positive elevation anomalies consistent with barrels; a photo-survey was conducted, showing multiple barrels on the seafloor. The ROV Jason was then deployed for additional images of the barrels, and to collect sediment samples for chemical measurements [previously described (Kivenson et al., 2019)], and biological analyses. The bottom waters are oxygen-limited (between 0 and 5 μM oxygen) and the sediments can support sulfate reduction (Dailey et al., 1993). The core samples used for biological analyses include the surficial (top two centimeters) sediment from two push cores not affiliated with barrels (collected >60 m from a barrel), two microbial mat/ring samples (collected at the microbial ring-shaped mat from each of two barrels), and two samples from just outside the microbial ring/mat (<0.25 m). Sediment samples for biological analyses were sectioned shipboard following ROV retrieval, and stored at −80°C prior to DNA extraction.

### DNA Extraction and Preparation

Six core-top (0–2 cm) sediment samples were processed for 16S rRNA genes and metagenomic sequencing. First, bulk DNA was extracted from ∼0.25 g of each sediment sample using a MoBio PowerSoil DNA extraction kit according to manufacturer instructions, with the following modification: bead beating (60 s) to enhance cellular lysis for greater DNA yield.

### 16S rRNA Gene Sequencing and Analyses

The V4 region of the 16S SSU rRNA gene was amplified using primers (515F-806R) optimized for sequencing on the Illumina MiSeq platform as described previously (Kozich et al., 2013; Apprill et al., 2015). Amplicon PCR reactions contained 1 μl template DNA (5 ng/μl), 2 μl forward primer, 2 μl reverse primer, and 17 μl AccuPrime Pfx SuperMix. Thermocycling consisted of 95°C for 2 min, 30 cycles of 95°C for 20 s, 55°C for 15 s, 72°C for 5 min, and a final elongation at 72°C for 10 min. A purification step using a Qiagen APure XP bead cleanup kit was performed. Sample concentrations were normalized using the SequelPrep Normalization Kit and visualized on an Agilent 2200 TapeStation with the genomic DNA ScreenTape at the California NanoSystems Institute at University of California, Santa Barbara. Samples were sequenced at the UC Davis Genome Center on the Illumina MiSeq platform with 250 nt, paired end reads. Raw sequences were quality checked and analyzed using the two open-source software packages: mothur v.1.36.1 for operational taxonomic units (OTUs) (Kozich et al., 2013) and DADA2 v.1.1.0 for amplicon sequence variants (ASVs) (Callahan et al., 2016, 2), using the SILVA bacterial reference database (Release 128) (Pruesse et al., 2007). The standard Miseq SOP pipeline using mothur (Kozich et al., 2013) was performed to enable comparison with another San Pedro Basin dataset referenced in the results, and the ASV pipeline using DADA2 was used in order to resolve fine scale sequence variation (Callahan et al., 2016, 2). Phyloseq was used to for visualization, to examine community diversity and ASV abundance (McMurdie and Holmes, 2013).

### Metagenomic Preparation and Sequencing

For metagenomic sequencing, following purification, DNA concentrations were determined using Invitrogen Qubit fluorometric quantification and normalized for library preparation (5 ng/μL). The Nextera XT library preparation kit was used in accordance with manufacturer instructions with custom Illumina indexes for demultiplexing. The concentration and size of each sample following library preparation were validated using the Qubit and Agilent 2200 TapeStation with the genomic DNA ScreenTape, respectively. Whole genome sequencing was performed at the California NanoSystems Institute at University of California, Santa Barbara using the Illumina NextSeq 500 platform, using v2 chemistry and the Mid Output flow cell kit, generating 78.8 GB of paired end 150 bp reads representing the collected sampled DNA.

### Genome Reconstruction From Whole Genome Sequencing Data

Quality control for reads was performed using Trimmomatic v.0.36 to remove sequencing artifacts and low quality reads (Bolger et al., 2014). Adapters and short reads were removed using the adaptive read trimmer, Sickle v.1.33 (Joshi and Fass, 2018). After quality control, reads for each sample were individually assembled using MetaSpades v.3.10.1 (Nurk et al., 2017). Additionally, Megahit v.1.1.2 assembly was generated for comparison and included in Supplementary Table 2 (Li et al., 2015). Default parameters were used for all software unless otherwise specified. The quality of the assemblies was determined using QUAST v.4.6 (Gurevich et al., 2013). Trimmed (post quality-control) sequencing reads for each sample were mapped to assembled contigs ≥1000 bp, using Bowtie2 v.2.3 and Samtools v.1.7 (Langmead et al., 2009; Li et al., 2009). GC content, tetranucleotide content, and coverage were used manually within Anvi’o v.6.2.0 to generate genome bins (Eren et al., 2015, 2021). The bins were manually refined using Anvi’o on the basis of consistency of coverage and nucleotide content of contigs in each bin, in order to reconstruct metagenome-assembled genomes (MAGs). Completion and redundancy for each refined MAG was determined with Anvi’o on the basis of recovery (completion) and repeats (redundancy) of conserved single copy genes of the nearest taxonomic group. Recovered MAGs are described following the genome reporting standards developed by the Genomic Standards Consortium (Supplementary Table 13; Bowers et al., 2017).

### Functional Annotation and Taxonomic Assignment of Genomes

Predicted proteins in each genome were identified using Prodigal v.2.6.3 individually (single genome mode) and functional annotation was performed using Hmmer v.3.1, with hmmscan (1E-10) with top hits only, against the Pfam database v.31 and Tigrfam database v.15.0 (Haft et al., 2003; Hyatt et al., 2010; Finn et al., 2011, 2013). Additional protein annotation was performed to assign proteins to COG categories using the rps-blast option in Blast v.2.7.1 (Altschul et al., 1990) and the Perl script cdd2cog, available at https://github.com/aleimba/bac-genomics-scripts/tree/master. The Phyre2 web portal was used for protein modeling and prediction (Kelley et al., 2015). RNAmmer v.1.2 was used for identification and recovery of 16S rRNA gene sequences from the reconstructed genomes (Lagesen et al., 2007). Taxonomy of reconstructed genomes was determined with preference given to criteria in the following order: (i) 16S rRNA genes, when recovered, (ii) recA, due to its strong phylogenetic signal, and (iii) taxonomic assignment using other ribosomal protein sequences (Rp_S8) (Table 1 and Supplementary Table 3). GTDB-TK v.1.3.0 was used to assign taxonomic identification using the RS95 database release, and is included for reference (Supplementary Table 4; Chaumeil et al., 2020), but established taxonomic nomenclature is used throughout the manuscript for consistency. Recovered genome-origin 16S rRNA sequences were aligned using Muscle v.3.8 with candidate ASV sequences from the 16S rRNA gene data set to determine the percent abundance of corresponding genomes (Edgar, 2004). To compare genomes from different samples, the average nucleotide identity (ANI) and average amino acid identity (AAI) were used to measure nucleotide level genomic similarity for genomes with high completion levels, using the Enveomics webserver ANI and AAI calculators (Rodriguez-R and Konstantinidis, 2016). Additional ANI and AAI values for Deltaproteobacteria- bbl genomes and their closest cultivated representatives are included (Supplementary Table 14).

### Characterization of GCE and Application of Non-canonical Protein Translation Tables

To identify instances of genetic code expansion in reconstructed genomes, the functional annotation results for genomes were compared with previously described cellular machinery required for the co-translational insertion of Sec and Pyl (Supplementary Table 12). Additionally, the requisite Sec- and Pyl- specific tRNA sequences, as well as their secondary structures and corresponding anticodons were identified using the Aragorn web server (Laslett and Canback, 2004). The anticodon CUA was identified for TAG stop-codon suppression and Pyl insertion, and the anticodon UCA was identified for TGA stop-codon suppression and Sec insertion, as well as the elongated extra arm of the Sec tRNA. The Sec-tRNA structure was further validated using the Secmarker tool via the webserver at https://secmarker.crg.es/ and Pyl-tRNA structure was confirmed using Infernal v.1.1.2 (Nawrocki and Eddy, 2013; Santesmasses et al., 2017). The secondary structures of each tRNA were also manually inspected against previously characterized Sec- and Pyl- tRNA structures.

For genomes with an expanded genetic code, alternate protein prediction methods were applied for identification and validation of repurposed TAG and TGA codons. For Pyl residue identification in proteins, the source code for Prodigal was modified to implement a custom translation table with in-frame TAG readthrough, as previously described (Kivenson and Giovannoni, 2020). The modified code and documentation for the TAG readthrough translation table is freely available at https://github.com/VeronikaKivenson/Prodigal. Next, protein sequence alignment was performed for proteins with in-frame TAG codons in the relevant methyltransferases using Muscle v.3.8 (Edgar, 2004). These alignments were then manually inspected to determine if the region containing and following the TAG codon in Deltaproteobacterial proteins is conserved as compared to pyrrolysine-containing methyltransferase protein sequences originating from archaea (Paul et al., 2000).

Proteins containing selenocysteine were identified and validated using a list of previously characterized selenoproteins (Zhang and Gladyshev, 2005, 2007, 2008; Zhang et al., 2005; Peng et al., 2016). These proteins as well as possible, novel selenoproteins were identified and analyzed using the program, bSECISearch, which applies a bacterial SECIS consensus model for the detection and identification of the SECIS element and the in-frame UGA sequence encoding Sec (Zhang and Gladyshev, 2005).

For PylB phylogenetic reconstruction, the MAG-derived proteins from this study were used to search for homologs, from which a non-redundant set of representatives was generated using CD-HIT (Li and Godzik, 2006), with a 90% global identity threshold. Next, sequences were aligned using MUSCLE (Edgar, 2004) and PylB protein trees were constructed using FastTree v.2.1.5 (Price et al., 2010) with the Whelan and Goldman model and optimized Gamma20 likelihood. For comparison, another PylB tree was constructed using RAXML v.8.2.12 (Kozlov et al., 2019), with the LG substitution matrix, and automatic determination of optimal bootstraps (Supplementary Figures 5, 6).

### Identification and Distribution of Specialized Deltaproteobacteria in the Environment

Publicly available metagenomic, genomic, and metatranscriptomic data sets were examined to determine the environmental distribution and niche preference of specialized Deltaproteobacteria with an expanded genetic code and augmented metabolism (Woyke et al., 2006; Pruitt et al., 2007; Hawley et al., 2014; Kyrpides et al., 2014; Smith et al., 2015, 2019; Thureborn et al., 2016; Descamps et al., 2017; Dombrowski et al., 2017, 2018; Dong et al., 2019; Supplementary Table 11).

A list of candidate GCE- capable Deltaproteobacterial genomes was compiled on the premise of ribosomal protein sequence similarity with Deltaproteobacterial-bbl genomes identified in this study (Supplementary Table 11). Annotation of these candidate genomes was performed to determine if they encode the requisite components for GCE, as described in the preceding section.

Short read datasets from select studies on oxygen-limited environments were analyzed due to their ability to host the most closely related and well-characterized genera: the *Desulfobacula*. The targeted locations included hydrothermal vents, oil seeps, anaerobic sediments, and other oxygen-limited and/or sediment environments. Sequencing reads were accessed from the NCBI SRA site on June 19, 2019, and downloaded using the SRA-dump option of the NCBI SRA Toolkit v2.8.1. These short-read data sets were individually assembled, and annotation of the resulting contigs and partial genomes was performed as previously described. Taxonomic assignment was determined using ribosomal and other protein sequences to identify and confirm Deltaproteobacterial contigs and partial genomes, and subsequent identification of GCE machinery and augmented metabolism was performed as previously described.

Protein searches were performed using the amino acid sequences of Deltaprotebacteria-bbl proteins that are central to GCE and augmented metabolism pathways from this study after candidate metatranscriptomic data sets were identified and processed as described below. The JGI IMG webserver^1^ was accessed on July 30, 2019 for data from the Genomes Online Database (GOLD) resource (Mukherjee et al., 2021); sediment environments were targeted due to the observation that such environments can host specialized Deltaproteobacteria with the relevant metabolism. This data further supplemented the data examined in the previous two steps, and led to the identification and analysis of metatranscriptomic data sets from sediments from geographic areas of interest. Following quality control, the assembly, protein prediction, and gene annotation steps were performed as previously described, with the addition of a secondary protein prediction step using FragGeneScan v.1.30 for identification of proteins in fragmented sequences in metatranscriptomic data (Rho et al., 2010).

ChemDraw and BioRender software were used for creating graphics for primary and supplementary figures. The computational biology data processing and analysis workflow was completed using the Extreme Science and Engineering Discovery Environment (XSEDE) Bridges resource at the Pittsburgh Supercomputing Center (Towns et al., 2014; Nystrom et al., 2015).

## Data Availability Statement

The sequences reported in this paper have been deposited in the National Center for Biotechnology Information Sequence Read Archive, www.ncbi.nlm.nih.gov/ (accession no. PRJNA726559).

## Author Contributions

VK, BP, and DV designed the investigation and collected the samples. VK prepared samples for sequencing, performed 16S rRNA gene and metagenome processing, metabolic reconstructions, and identified re-purposed stop codons. VK and BP identified and analyzed genetic code expansion machinery. All authors contributed to manuscript preparation.

## Conflict of Interest

The authors declare that the research was conducted in the absence of any commercial or financial relationships that could be construed as a potential conflict of interest.

## Footnotes

1 https://gold.jgi.doe.gov
